# MBCdeg4: A modified clustering-based method for identifying differentially expressed genes from RNA-seq data

**DOI:** 10.1016/j.mex.2024.103149

**Published:** 2024-12-30

**Authors:** Chiharu Ichikawa, Koji Kadota

**Affiliations:** a Graduate School of Agricultural and Life Sciences, The University of Tokyo, Yayoi 1-1-1, Bunkyo-ku, Tokyo 113-8657, Japan; b Interfaculty Initiative in Information Studies, The University of Tokyo, Hongo 7-3-1, Bunkyo-ku, Tokyo 113-0033, Japan; c Collaborative Research Institute for Innovative Microbiology, The University of Tokyo, Yayoi 1-1-1, Bunkyo-ku, Tokyo 113-8657, Japan

**Keywords:** Gene expression, Normalization, Gene clustering, R package, MBCdeg4

## Abstract

RNA-seq is a commonly employed methodology for the measurement of transcriptomes, particularly for the identification of differentially expressed genes (DEGs) between different conditions or groups. In a previous report, we described a clustering-based method for identifying DEGs, designated MBCdeg1 and MBCdeg2. and a modified version, MBCdeg3. This study presents a further improved version, designated MBCdeg4. The four versions of MBCdeg employ an R package, designated MBCluster.Seq, internally. The sole distinction between them is the manner of data normalization. MBCdeg4 employs normalization factors derived from a robust normalization algorithm, designated as DEGES. Seven competing methods were compared: the four versions of MBCdeg and three conventional R packages (edgeR, DESeq2, and TCC). MBCdeg4 demonstrated superior performance in a multitude of simulation scenarios involving RNA-seq count data. Therefore, MBCdeg4 is recommended for use in preference to the earlier versions, MBCdeg1–3.

• MBCdeg4 is a method for both identification and classification of DEGs from RNA-seq count data.
• MBCdeg4 is available as an R function and performs well in a wide variety of simulation scenarios.

## Graphical abstract

**Figure:**
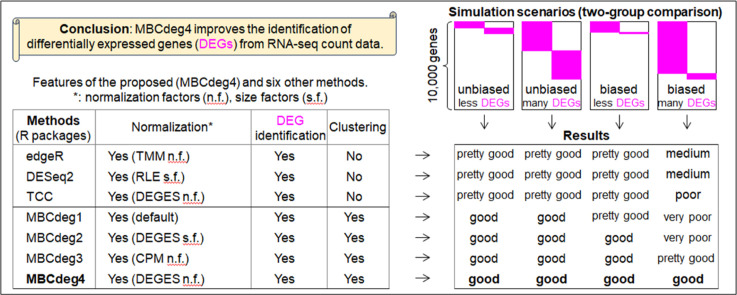

Specifications table

**Table:** 

Subject area:	Bioinformatics
More specific subject area:	Gene expression analysis
Name of your method:	MBCdeg4
Name and reference of original method:	MBCdeg1 and 2: T. Osabe, K. Shimizu, K. Kadota, Differential expression analysis using a model-based gene clustering algorithm for RNA-seq data, BMC Bioinformatics, 22 (2021) 511. 10.1186/s12859-021-04,438-4 MBCdeg3: M. Makino, K. Shimizu, K. Kadota, Enhanced clustering-based differential expression analysis method for RNA-seq data, MethodsX 12 (2023) 102,518, 10.1016/j.mex.2023.102518
Resource availability:	MBCdeg's original GitHub site https://github.com/takosa/MBCdeg-paper/ (last access: October 28th, 2024)

## Background

The identification of DEGs for different groups or conditions represents one of the most common reasons for analyzing RNA-seq data [[1], [2], [3]]. The process of differential expression (DE) analysis typically commences with the utilization of a numerical matrix, designated as “count data”, wherein the rows represent genes and the columns represent samples. The outcome of this analysis is the assignment of *p*- and/or *q*-values assigned to individual genes [[4], [5], [6], [7]]. In previous work, we have proposed a clustering-based DE algorithm (referred to as MBCdeg1 and 2) [8] and a modified version of this (referred to as MBCdeg3) [9]. The algorithm employs an R package, MBCluster.Seq [10], which is dedicated to gene clustering. Consider applying K-means clustering with K = 3 to count data comparing two groups (G1 vs. G2). It is reasonable to expect that the resulting expression patterns of the three cluster centers will exhibit the following characteristics: (i) up-regulated in G1 ("DEG1″ pattern), (ii) up-regulated in G2 ("DEG2"), and (iii) consistent in both groups ("non-DEG"). The lower the posterior probability (PP) assigned to the “non-DEG” cluster, the higher the degree of DE for that gene [8]. As demonstrated by other researchers and ours, the clustering-based DE analysis methods simultaneously identify DEG and perform gene clustering [8,11].

The three versions of MBCdeg, which employ different normalization algorithms, utilize count data with a pre-specified number of clusters, K, to generate representative expression patterns for individual clusters and cluster numbers with the PP for individual genes. MBCdeg1 employs the default normalization algorithm embedded within MBCluster.Seq, to adjust the upper quartile (UQ) of the counts for all genes across samples. MBCdeg2 employs the DEGES normalization algorithm [12], as implemented in the R package TCC [13], to adjust the trimmed mean of the counts of potential non-DEGs. MBCdeg3 employs the counts per million (CPM) normalization, to adjust the mean counts of all genes across samples. MBCdeg1 and 3 employ normalization factors obtained from UQ or CPM, whereas MBCdeg2 employs size factors obtained from DEGES normalization factors.

The terms “size factors” and “normalization factors” are employed in two prominent packages, DESeq2 [14] and edgeR [15], respectively. In edgeR, the effective library size is calculated by multiplying the library size by the normalization factor. In DESeq2, the size factors are comparable to the *normalized* effective library sizes, wherein the summary statistics for the effective library sizes are adjusted to one [13]. Our recent analysis of MBCdeg3 has led us to hypothesize that the use of DEGES normalization factors in lieu of size factors may prove beneficial in terms of performance. In this paper, we present an improved pipeline, designated MBCdeg4, which employs DEGES normalization factors in the MBCdeg algorithm.

### Method details

The methodology for identifying DEGs based on gene clustering is outlined in our previous paper [8]. The sole distinction between the four versions of MBCdeg lies in the normalization phase. Accordingly, we have employed notations analogous to those described in [8] for consistency. In this study, an input count matrix is defined as a matrix where each row represents a gene (g = 1, …, G), each column represents a replicate (*j* = 1, …, *n_i_*) of group (*i* = 1, …, *I*), and each cell represents the number of counts. In this context, G represents the number of genes, *I* denotes the number of groups being compared, and *n_i_* is the number of replicates for the group *i*. MBCdeg clusters gene vectors (*β*_g_ = *β_g_*_1_, …, *β_gI_*), where *β_gi_* indicates the count of gene g in the group *i* relative to the overall mean on a log-scale. Consequently, the sum of *β_gi_* for a given gene g across all compared groups will be equal to zero. *β*_g_ can be considered as the log fold-change (*FC*) between the compared groups.

In response to a specified cluster number (K), MBCdeg returns two results as output. One result is the center for cluster *k, μ_k_* = (*μ_k_*_1_, …, *μ_kI_*) for *k* = 1, …, K, and the other is the PP that gene g belongs to the *k*^th^ cluster, *p_g_* = (*p_g_*_1_, …, *p_gK_*) for g = 1, …, G. To identify the non-DEG cluster, MBCdeg considers the *L*^2^ Norm of *μ_k_* for each cluster center across groups, ||*μ_k_*||_2_ = (|*μ_k_*_1_|^2^ + … + |*μ_kI_*|^2^)^1/2^: A smaller value of the norm for cluster *k* indicates a lower degree of DE across groups for that cluster. Therefore, one can consider the probability of a gene being located in the *k*^th^ column as being in the non-DEG cluster, where *k* = argmin(||*μ*_1_||_2_, …, ||*μ*_K_||_2_). A lower value of the PP for gene g in the *k*^th^ cluster (i.e., *p_gk_*) indicates a higher degree of DE between the compared groups [8].

**Fig. 1:**
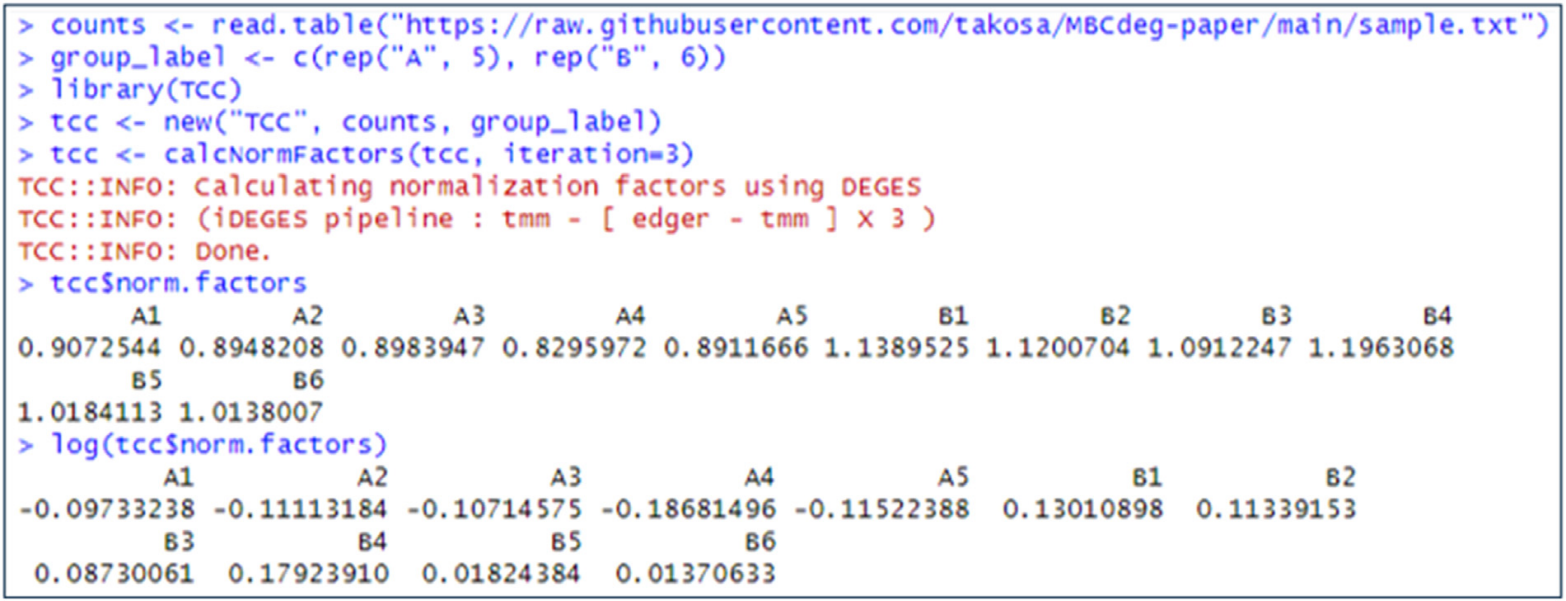
Calculation of the DEGES normalization factors.

In practical implementation, MBCdeg4 employs the logarithm of the DEGES normalization factors, which can be calculated using TCC [13]. Fig. 1 illustrates an example of the calculation of “log(DEGES normalization factors)” from a simulation count dataset provided on Osabe's GitHub page (https://raw.githubusercontent.com/takosa/MBCdeg-paper/main/sample.txt). The dataset comprises 2000 genes and 11 samples, with five Group A and six Group B samples (labeled “A1”, …, “A5”, “B1”, …, “B6”). This results in G = 2000, *I* = 2, *n_A_* = 5, and *n_B_* = 6. As this dataset is identical to that used in [9], the current results using this dataset can be compared with those for MBCdeg3. In contrast to the CPM normalization, the calculation of the DEGES normalization factors requires the group label information. Utilizing this information, TCC is capable of identifying potential DEGs between groups undergoing comparison. The elimination of potential DEGs is essential to obtain good normalized data. The DEGES normalization factors calculated from the potential non-DEGs can, at least in theory, increase both sensitivity and specificity for identifying DEGs [12].

**Fig. 2:**
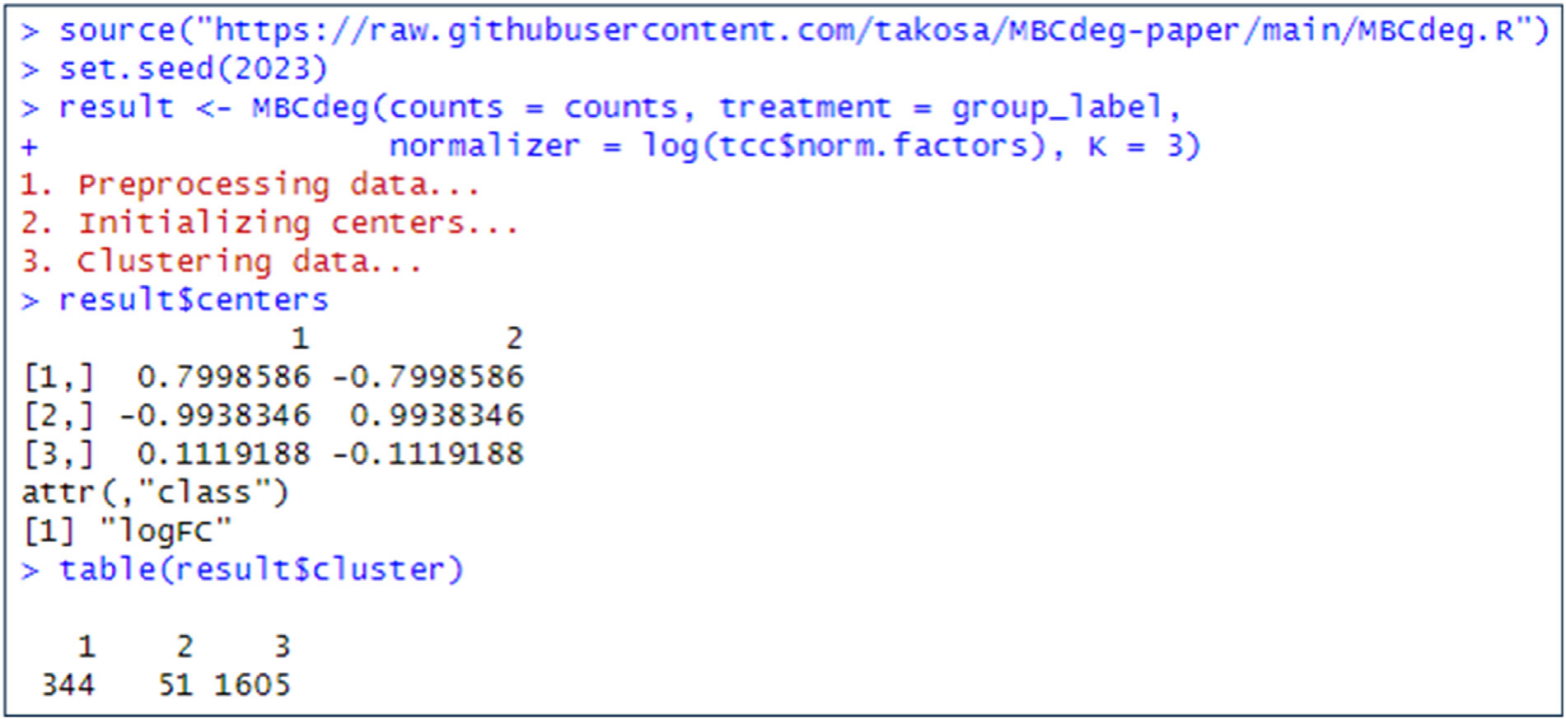
Execution of MBCdeg4.

Fig. 2 illustrates the execution of MBCdeg4 with the dataset. A wrapper function, designated “*MBCdeg*”, was employed [8], and this function was sourced from Osabe's GitHub page (https://raw.githubusercontent.com/takosa/MBCdeg-paper/main/MBCdeg.R). This function takes four arguments in total: the raw count data, the group label information, the log-transformed scaling factors (in this case, the DEGES normalization factors), and the preselected number of clusters, K. For this dataset, MBCdeg4 with K = 3 returns the centers for cluster *k* (=1, 2, 3) as follows: *μ*_1_ = (0.80, −0.80) with 344 genes, *μ*_2_ = (−0.99, 0.99) with 51 genes, and *μ*_3_ = (0.11, −0.11) with 1605 genes. The results indicate that *μ*_1_ and *μ*_3_ exhibit the DEG1 pattern, while *μ*_2_ exhibits the DEG2 pattern. In this instance, the third cluster with the smallest norm (||*μ*_3_||_2_ = 0.158) is identified as the non-DEG cluster and the PP values are employed for gene ranking.

### Method validation

The dataset comprises 2000 genes, of which 20 % are DEGs and the remaining 80 % are non-DEGs. The DEG1 pattern is observed in 90 % of the DEGs, representing 360 genes. This pattern is characterized by a four-fold increase in Group A. The remaining 10 % (i.e., 2000 × 0.2 × 0.1 = 40 genes) exhibit the DEG2 pattern (up-regulated 9-fold in Group B). The proportion of DEGs is denoted as *P_DEG_*, while the proportion of genes that are up-regulated in Group A is denoted as *P_A_*. Accordingly, the simulation scenario corresponds to *P_DEG_* = 0.2 and *P_A_* = 0.9. The DEG1 pattern is up-regulated in 4-fold in A, indicating that the ideal *μ*_1_ is (0.69, −0.69), with log_e_(4^1/2^) = 0.69. Similarly, the DEG2 pattern, which was up-regulated in 9-fold in B, indicates that the ideal *μ*_2_ is (−1.1, 1.1), with log_e_(9^1/2^) = 1.1. In contrast, the non-DEG pattern, which was up-regulated in 1-fold in both groups, suggests that the ideal *μ*_3_ is (0, 0), with log_e_(1^1/2^) = 0. As illustrated in Fig. 2, the actual results obtained with MBCdeg4 are more similar to the ideal values than those obtained with MBCdeg3 (Fig. 2 of [9]). The area under the receiver operating characteristic curve (AUC) was calculated using the ranked gene list and the corresponding list of true DEGs or non-DEGs, resulting in an AUC value of 0.9802. This value is similar to that observed for MBCdeg3 (Fig. 3 of [9]).

**Fig. 3:**
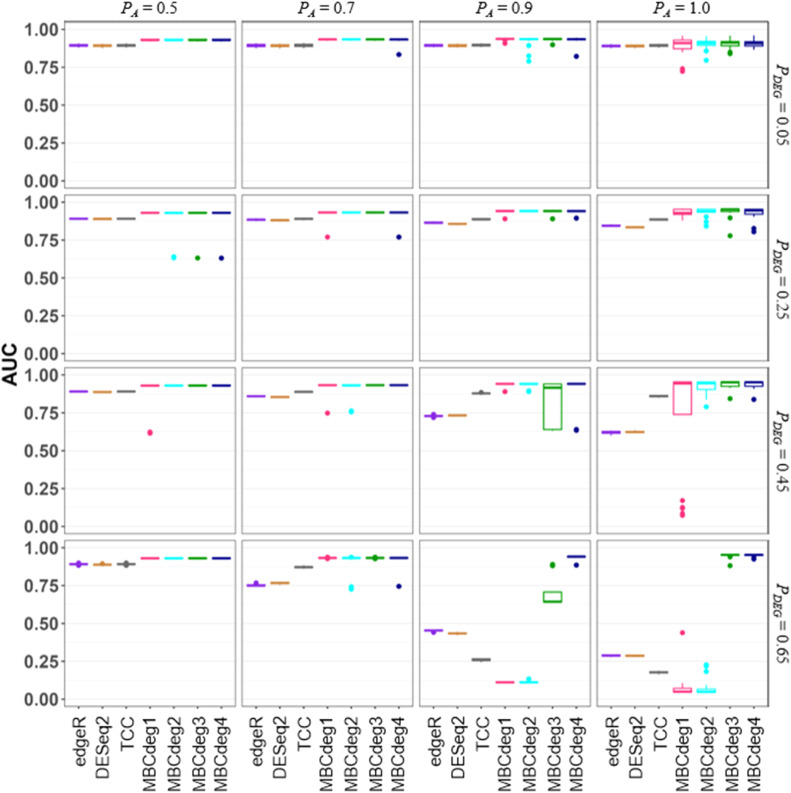
Simulation results (lower *P_DEG_* values). Boxplots of the AUC values (20 trials) for each method under a total of 16 conditions, *P_A_* = 0.5 (left) to 1.0 (right) with *P_DEG_* = 0.05 (top) to 0.65 (bottom), are shown.

Subsequently, the results are presented, which compare a total of seven competing methods/packages (edgeR, DESeq2, TCC, and MBCdeg1–4) under different simulation conditions. In accordance with the preceding study, we present the performance of these methods based on the identical evaluation metric and simulation framework [9]. The AUC is employed as a comparison metric, which evaluates both sensitivity and specificity of the methods simultaneously. The simulation conditions are as follows: (i) G = 10,000, (ii) *I* = 2 and *n_A_* = *n_B_* = 3, (iii) *FC* = 4, (iv) *P_DEG_* = 0.05, 0.25, 0.45, and 0.65, and (v) *P_A_* = 0.5, 0.7, 0.9, and 1.0. A higher *P_A_* value indicates a higher degree of up-regulated DEGs in Group A, ranging from unbiased (*P_A_* = 0.5) to fully biased (*P_A_* = 1.0) conditions. Fig. 3 depicts the AUC values for the seven methods across a total of 16 simulation conditions, with 20 trials conducted for each condition. It should be noted that the values, with the exception of MBCdeg4, are essentially identical to those presented in our previous study (see Fig. 4 of [9]). In general, the performance of MBCdeg4 is satisfactory. While the AUC values of MBCdeg3 at *P_A_* = 0.9 tend to be lower than in other *P_A_* conditions, MBCdeg4 is able to compensate for this shortcoming.

**Fig. 4:**
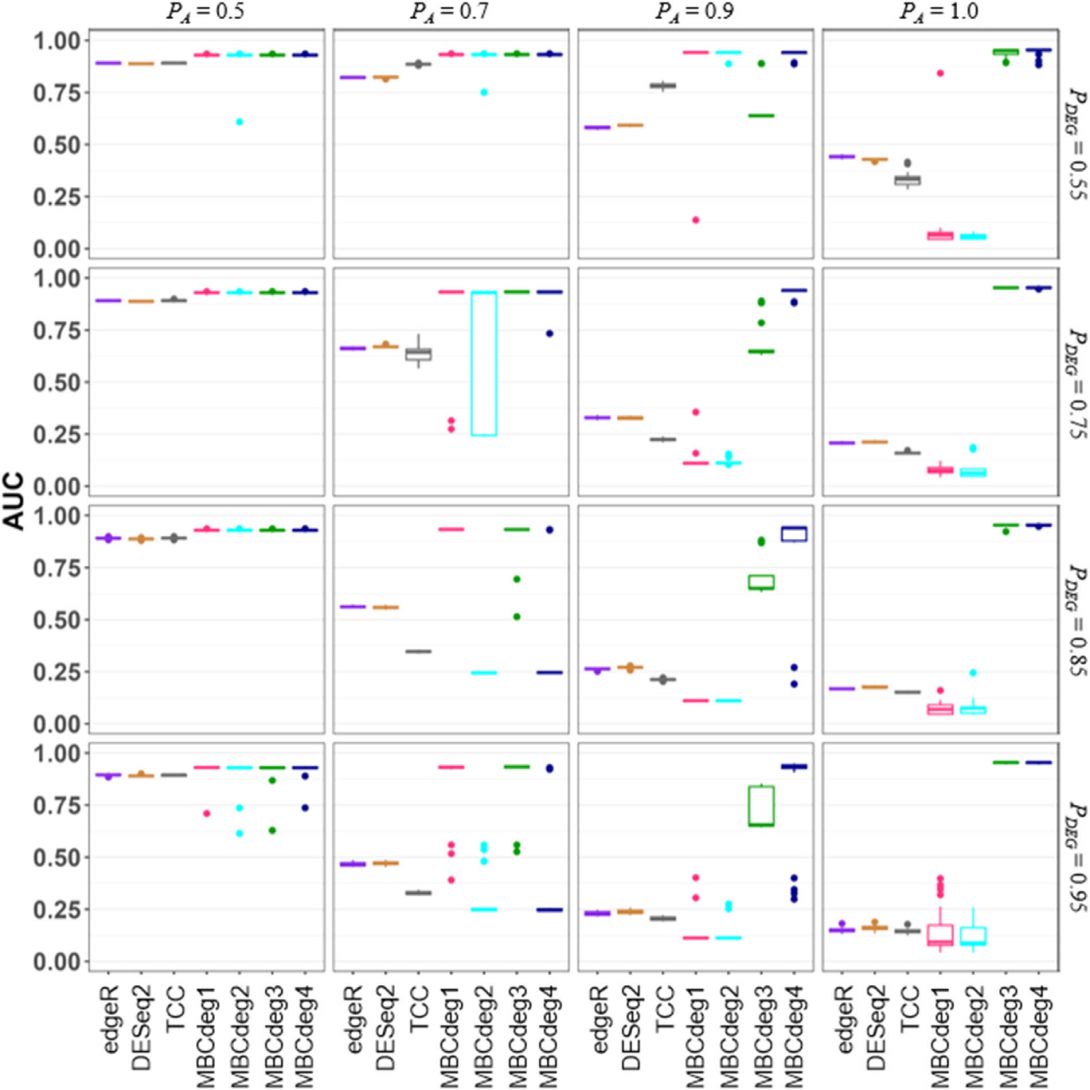
Simulation results (higher *P_DEG_* values).

### Limitations

It is necessary to illustrate the constraints of MBCdeg4. Fig. 4 illustrates the AUC values for *P_DEG_* values of 0.55, 0.75, 0.85, and 0.95. As the other conditions are identical to those in Fig. 3, a collective discussion of the results is warranted. It is evident that MBCdeg4 is markedly inferior at *P_DEG_* ≥ 0.85 and *P_A_* = 0.7, and that MBCdeg4 exhibits exceedingly low AUC values in certain trials at *P_DEG_* ≥ 0.85 and *P_A_* = 0.9. As previously discussed in [8], the majority of these markedly low AUC values can be attributed to misidentification of the non-DEG cluster. Furthermore, while the majority of trials exhibit high AUC values, a few trials demonstrate slightly lower AUC values. For example, MBCdeg4 demonstrated such a result (AUC = 0.73) at *P_DEG_* = 0.75 and *P_A_* = 0.7. As previously discussed in [8], the majority of these relatively low AUC values are primarily attributable to the incorporation of the DEG1 and/or DEG2 patterns within the non-DEG cluster. These findings suggest that MBCdeg4 is not a universal method for all scenarios.

It is our understanding that there is no other method that can successfully rank genes in a wide range of simulation scenarios, from relatively simple conditions (e.g., *P_DEG_* = 0.05 and *P_A_* = 0.5) to challenging conditions (*P_DEG_* = 0.95 and *P_A_* = 1.0). The success of MBCdeg4 can be attributed to two reasons. Firstly, all versions of MBCdeg demonstrate robust performance at relatively low *P_DEG_* values (< 0.5), where discrepancies in normalization methods, including the selection of scaling factors, normalization factors (MBCdeg1, 3, and 4) or size factors (MBCdeg2), are inconsequential. Secondly, the incorporation of normalization factors is, in fact, an erroneous approach when utilizing MBCdeg. It is our hypothesis that MBCluster.Seq, which is utilized internally by MBCdeg, was originally designed to be provided with the size factors in lieu of the normalization factors.

**Fig. 5:**
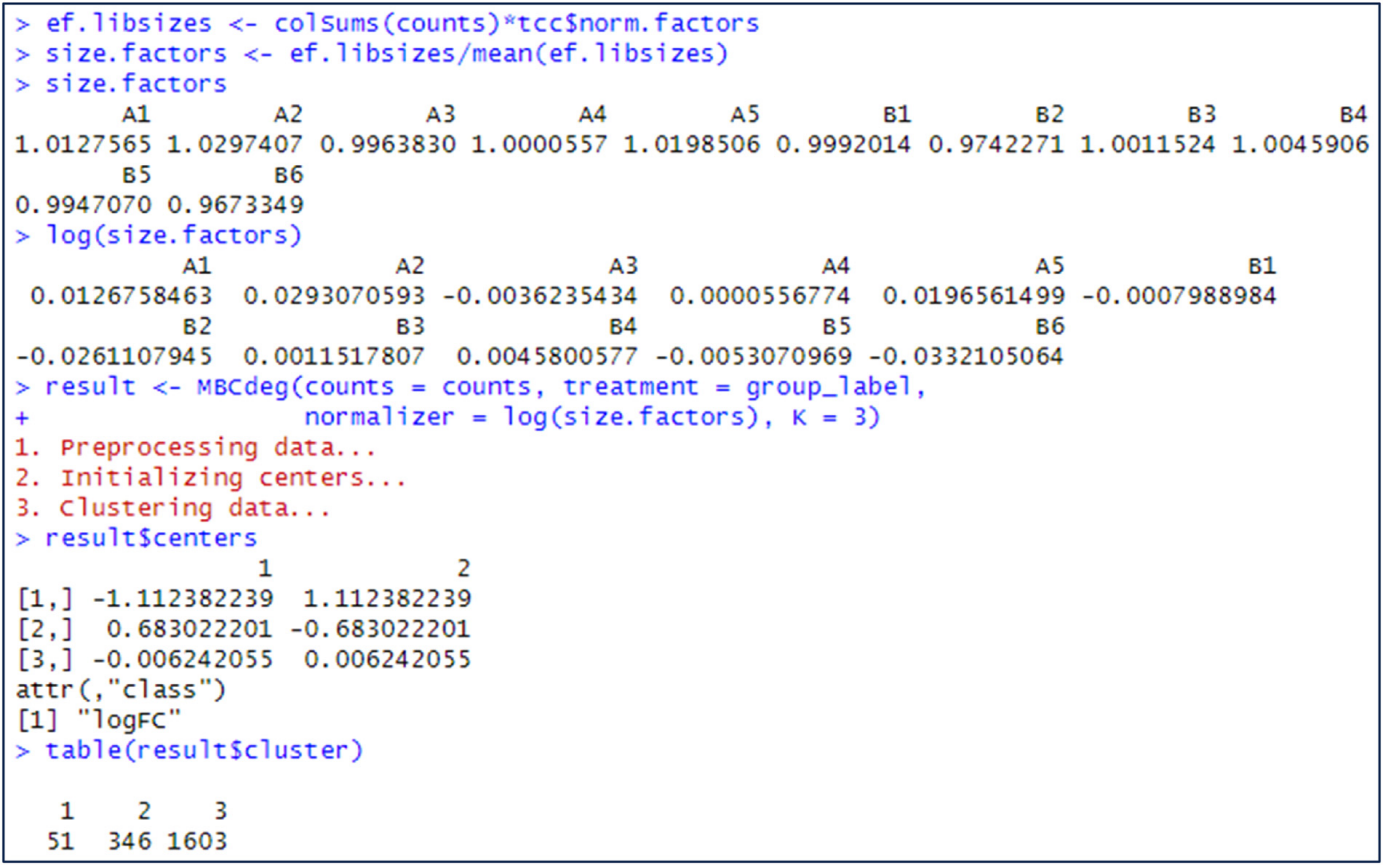
Calculation of the DEGES size factors and execution of MBCdeg2. The AUC value is 0.9802 as high as that of MBCdeg4.

Fig. 5 illustrates the calculation of the DEGES size factors from the normalization factors, accompanied by the outcome of MBCdeg2 for the identical dataset utilized in Figs. 1 and 2. The results demonstrate that the cluster centers are *μ*_1_ = (−1.11, 1.11) with 51 genes, *μ*_2_ = (0.68, −0.68) with 346 genes, and *μ*_3_ = (−0.01, 0.01) with 1603 genes. This indicates that *μ*_1_ and *μ*_2_ exhibit the DEG2 and DEG1 patterns, respectively, while the third cluster, which has the smallest norm (||*μ*_3_||_2_ = 0.009), is identified as the non-DEG cluster. It is evident that the expression patterns for the cluster centers derived from MBCdeg2 are more similar to the ideal patterns than those derived from MBCdeg4 (Fig. 2). Note that the size factors are, on the whole, higher in Group A than in Group B (Fig. 5), and that the values between the groups are opposite in the normalization factors (Fig. 1). This indicates that the two types of factors are analogous in that they are employed as scaling factors in the normalization step, but their effects on each group are markedly disparate. This provides support for the claim that the appropriate scaling factors to be applied to the MBCdeg are size factors, rather than normalization factors. It is thus recommended that MBCdeg2 be employed in lieu of MBCdeg4 when the true DEG is known to be <50 %.

The superior performance of MBCdeg4 in challenging conditions, such as *P_DEG_* ≥ 0.65 and *P_A_* ≥ 0.9, can be attributed to the markedly inferior performance of MBCdeg2. The performance distribution of MBCdeg2 is analogous to that of the DEGES algorithm itself, as evidenced in [16]. This suggests that MBCdeg will perform well in conjunction with any scaling factors that exert an inverse effect on the DEGES size factors under such conditions. The employment of DEGES normalization factors in MBCdeg4 serves to exemplify this concept. Although the concept may initially appear somewhat heuristic, it can be confirmed that MBCdeg3 incorporates the CPM normalization factors. This study makes an important contribution to the field by providing a logical rationale for the exceptional performance of MBCdeg3 and 4 in challenging conditions.

Given the markedly inferior performance of MBCdeg4 at *P_DEG_* ≥ 0.85 and *P_A_* = 0.7 (see Fig. 4), it is conceivable that certain researchers may reach the conclusion that there has been no substantial enhancement of MBCdeg4 over MBCdeg3. However, it is important to note that performance evaluation is not typically conducted under conditions of such extreme *P_DEG_* values. Instead, it is conducted under conditions of lesser severity, such as *P_DEG_* ≤ 0.75, in the majority of cases [8,9,17]. Therefore, it is imperative for the reader to understand the conditions under which the performance evaluation was conducted.

It is also important to note that the primary competitors of MBCdeg4 are MBCdeg1–3. This is due to three reasons. Firstly, TCC corresponds to an iterative and robust version of edgeR and/or DESeq2 [18]. Secondly, MBCdeg1 and 2 have demonstrated superior performance to TCC under the conditions where TCC performed as well as or better than the typical packages (edgeR and DESeq2) [8]. Thirdly, the current simulation framework is consistent with that employed in our previous study [8,9]. Although a direct comparison with real data has yet to be conducted, it seems reasonable to posit that a method demonstrated to be ineffective in a particular simulation scenario is unlikely to yield optimal results when applied to the real dataset in a similar or identical scenario. The findings of the present study suggest that MBCdeg4 is endorsed for utilization in preference to these earlier versions (MBCdeg1–3).

## Ethics statements

None

## CRediT author statement

**Chiharu Ichikawa:** Execution, Visualization, Investigation, Reviewing. **Koji Kadota:** Methodology, Confirmation, Writing- Reviewing and Editing.

## Declaration of competing interests

The authors declare that they have no known competing financial interests or personal relationships that could have appeared to influence the work reported in this paper.

## Data Availability

The data and codes are available on Supplementary materials.
